# Suggestions Towards a More Perfect System of Self-Acting Ventilation

**Published:** 1859-01

**Authors:** J. N. Radcliffe

**Affiliations:** late of the Staff of H.H. Omar Pacha.


### ORIGINAL COMMUNICATIONS.

### SUGGESTIONS TOWARDS A MORE PERFECT SYSTEM OF SELF-ACTING VENTILATION.

By J. N. RADCLIFFE, M.R.C.S., late of the Staff of H.H. Omar Pacha.

THE circumstances which gave rise to the suggestions which form the subject of this paper, arose mainly during the Crimean war. At that time, the question was often forced upon military medical men, how best to ventilate crowded huts, so that a sufficient amount of fresh air might be introduced into them, and the discomforts of currents avoided. The huts first sent out to the troops were ventilated chiefly by a window placed at each extremity of the hut, about six feet above the ground, and by the door. Subsequently huts of a better construction were erected, in which the windows were more numerous, and outlets for foul air were provided at the summit, and inlets for fresh air near the ground, generally beneath the platforms upon which the men reposed. Frequently, however, the outlets in the roof were too small to serve sufficiently the purposes for which they were intended ; frequently, also, both outlets and inlets were tampered with by the men, and stopped with rags.

When, as in the hospital huts, the foul air outlets were of sufficient magnitude and care was taken that the inlets were not interfered with, the ventilation was, upon the whole, satisfactory. But still, during the winter months, difficulties often occurred from, and injurious effects not unfrequently were produced by the occlusion of both inlets and outlets of air by the men, on the plea of discomfort from cold draughts. The Army Sanitary Commissioners in their report state this plea in the general form "cold weather," and consider that it was not a valid one. My experience led me to the conclusion that the plea, as I have expressed it, and have usually heard it expressed by the men, was valid, and that it was one of the most obnoxious discomforts in cold windy weather of the less sheltered, and particularly the more flimsily built huts. But be that as it may, it is certain that the men often stopped up the ventilating openings in the huts, and that, not unfrequently, evils arose from this source; and it was evident that, if a system of ventilation could be invented, by which a sufficiency of fresh air could be introduced into the huts, without occasioning the discomforts arising from draughts, and which, also, should be little likely to be tampered with, a great advantage would be obtained, both for the comfort and well-being of the soldiers.

Moreover, the solution of the question would most probably solve a problem of wider application than the ventilation of camp huts.

The Turkish soldier is much more addicted to close all means of egress or ingress of air into the hut, or tent, or building that he may be quartered in during cold weather, than the British soldier. Few greater sanitary evils can be found during a winter campaign in the Turkish army, than the dense crowding together of the Turkish soldiers in their quarters, and their persevering efforts to shut out fresh air.

Consequently, the evils arising from a want of a system of ventilation in huts, or quarters, which could not well be tampered with by the men, were more conspicuous in the Turkish than in the British army.

The only opportunity which I had during* the war of attempting to overcome the difficulty of obtaining a free, and yet not troublesome ventilation under circumstances similar to those stated, occurred, not in camp, but in quarters at Sinope.

A serious outbreak of fever had appeared on board a Turkish line-of-battle-ship in the harbour, and the sick had been removed into a temporary hospital on shore. The outbreak had been occasioned by the whole of the crew, upwards of five hundred men, being allowed to huddle together in the lower deck, for several nights during a short period of severe weather; one only of the hatches being open. The fever had been aggravated on shore by the sick being crowded as closely as they could be packed, into several small rooms; every inlet by which fresh air could obtain admission, and every outlet by which foul air could obtain egress, being closed. The sick, soon after being removed on shore, were consigned to the care of the medical staff, of which I was a member. Many of the men had been placed in rubble-built and tile-roofed sheds; and these men, to all intents and purposes, were circumstanced, as far as ventilation was concerned, similarly to the men quartered in huts in the field. In one of the sheds which was situated beneath the lofty wall of the inner quadrangle of the citadel, thirteen patients had to be accommodated, and their beds nearly filled the floor. The shed was lit by two small windows, the sashes of which were fixed, and the only opening either for the ingress or egress of air was the door. A wood ceiling was interposed between the tiled and sloping roof of the shed and the floor, at a height of about ten feet from the

* A report of this outbreak may be found in the Transactions of the Epidemiological Society for 1858, p. 13.

latter. Under these circumstances, and knowing the habits of the men, it was certain that any attempt to obtain a sufficient supply of fresh air from the door, or the windows, or the walls of the building would be useless; as, the moment the back of the medical man was turned, the nights and days being somewhat chilly, the openings would be closed. It had occurred to me several times in the Crimea, that the only system of ventilation which was adapted to circumstances of this kind, was the self-acting roof, or so-called siphon ventilator. If a tube or shaft, having a longitudinal diaphragm, communicate with a chamber containing air of a temperature higher than that of the air external to it, a double current will be established; a current of warm air flowing out of the chamber through one division of the tube, a current of cold air flowing into the chamber through the other division. I had had several opportunities of witnessing the success of this principle of ventilation in several large public rooms in England; and I determined, with the consent of the inspector of our staff, to give it a trial here. I forthwith caused capacious openings to be made in the ceiling and roof, and above this opening in the roof, a shaft was erected, several feet in height. The shaft was divided by a longitudinal partition within, and above it was erected a roof large enough to prevent the intrusion of wind or rain. The doors and windows were left untouched, and the ventilation was allowed to depend upon the roof shaft alone. Nothing could be more satisfactory than the result, for although, subsequently, it became necessary to place two or three beds more in the shed, and literally to cram it, the ventilation was most effective, and the patients did not complain of the slightest discomfort. In another shed the result was equally satisfactory; in a third, the ventilation failed in part, from the insufficient size of the openings.

Here then, in this system of ventilation, seemed to be a solution of the question respecting the comfortable and secure ventilation of crowded huts; and not of huts or sheds only, for several times in England, I had witnessed difficulties with regard to the ventilation of hospital wards and other rooms, similar to those encountered in the Crimean huts, and in the sheds at Sinope; but the necessity had never before been forced so fully upon me of overcoming them.

I remembered, however, that objections to the adoption of the siphon system of ventilation, except in lofty rooms, had been urged, from the upper stratum of air being kept at an uncomfortably diminished temperature, and from the prevalence of downward draughts in the rooms. Neither of these discomforts, so far as I could ascertain, occurred in the sheds at Sinope; and, considering the largeness of the ventilating shafts, and the lowness of the sheds, I was puzzled at first to account for this. At last, however, and after having again had my attention called to the practical use of this system of ventilation, it occurred to me that the ceiling in the sheds at Sinope played a more important part in obviating the objections alluded to than I had at first supposed. The interval between the ceiling and the tiles in the sheds constituted a large air-chamber, and the ceiling acted as a diaphragm between the air-chamber and the room. Was it not possible that the ceiling might have had the effect of diminishing the force with which the current of air from the shaft entered the shed, and also have prevented a too rapid reduction of temperature in the upper stratum of air? I am very much inclined to believe now, that this was the case, and that, by working out this conception, the application of the roof system of self-acting ventilation may be more widely extended.

In the instances in which I have seen the siphon system of ventilation carried most fully out, the shafts have been made to communicate directly with the room ventilated. In large and lofty public rooms, this perhaps might be an advantage, save and except that the amount of ventilation is usually made to depend upon the ornamental design of the architect, rather than upon the necessities of the building, several small perforated spaces in the ceiling being alone yielded for the work of ventilation. Again, the ventilators themselves have not been easy of architectural management, and most frequently they have disfigured the roof of the building externally. Now, I believe that my notions of a diaphragm might aid a little in bringing about that blessed epoch in architectural art, when architect and ventilating engineers might work with one heart and soul; and this, be it understood, in addition to removing the objections I have heard attached to the siphon system. Above all, I believe it would enable the system to be carried out to that liberal extent which is necessary to secure its efficiency without annoying either inmate or architect. For one of the greatest evils in all systems of ventilation is a niggardliness in providing both inlets and outlets of air.

Suppose that it were required to ventilate the wards of a two or three storied hospital, constructed in detached wings, the ventilation of each ward to be complete in itself; the self-acting ventilating shafts of the upper wards should be arranged in one or more rows upon the roof according to the requirements of the ward, the shafts being contained in the false roof, and their lower terminations opening into it. At a sufficient distance from the lower extremities of the shafts the ceiling should extend and this should be perforated freely, and in such ornamental designs as might be desired (affording no small scope for the decorative genius of the architect). The ceiling would act as a diaphragm to break and diffuse the currents of air, and it would also prevent the rapid reduction of the temperature of the apartment by a free introduction of fresh air from above. The arrangement of the shafts of the ventilators in the roof, would enable the architect to luxuriate in high pitched, double, ornamental roofs. If the ward had windows on both sides, and was warmed with stoves (for it is doubtful whether an open fire place would be consistent with self-acting roof ventilation) as perfect a ventilated apartment would exist as could be desired; for if the roof ventilation came to a stand-still, from the temperature of the outer and inner air approximating, the windows would then come into use as ventilators; and in winter the self-acting roof ventilation would be the more effective the closer the windows and doors were shut, and the more fully the stoves were in action. Not any, or but slight, discomforts would be experienced under these circumstances, by patients or attendants, from erratic draughts flowing from open windows or doors, or from inlets for the outer air near the floor, and still sufficient change of air might be obtained for all sanitary purposes. The same principle might be carried out in the lower wards by making an air chamber between the ceiling and the floor above, and fixing the ventilating shafts obliquely within it, so as to open upon the external wall, by ornamental perforated work. This would not necessitate so great an increase in actual loftiness of wards as might at first be supposed, because from the use to which the ceiling would be put it would not require to be placed at that height above the floor at which it is now most commonly placed.

By the use of a diaphragm and *quasi* air chamber, as in the above example, I believe that any objections which have been attached to the self-acting roof system of ventilation, may be in great measure avoided and its usefulness extended. This system seems to me well worthy of consideration, when in building hospitals or other public buildings, factories, etc., simplicity and inexpensiveness of style is required with effective ventilation, and it is probable that this system may be of more extensive application than is commonly supposed. In large cellular buildings, as gaols and lunatic asylums, a forced system of ventilation, plenum or vacuum, must usually be had recourse to; but there are many smaller buildings where the self-acting system of roof ventilation, in addition to the doors and windows, would probably meet every sanitary want, and remove or very greatly diminish the discomforts arising from draughts in boisterous, tempestuous, and cold weather.

